# Culture-Independent Investigation of the Microbiome Associated with the Nematode *Acrobeloides maximus*

**DOI:** 10.1371/journal.pone.0067425

**Published:** 2013-07-22

**Authors:** Jean-Paul Baquiran, Brian Thater, Sammy Sedky, Paul De Ley, David Crowley, Paul M. Orwin

**Affiliations:** 1 Department of Environmental Sciences, University of California Riverside, Riverside, California, United States of America; 2 Department of Nematology, University of California Riverside, Riverside, California, United States of America; 3 Department of Biology, California State University San Bernardino, San Bernardino, California, United States of America; J. Craig Venter Institute, United States of America

## Abstract

**Background:**

Symbioses between metazoans and microbes are widespread and vital to many ecosystems. Recent work with several nematode species has suggested that strong associations with microbial symbionts may also be common among members of this phylu. In this work we explore possible symbiosis between bacteria and the free living soil bacteriovorous nematode *Acrobeloides maximus*.

**Methodology:**

We used a soil microcosm approach to expose *A. maximus* populations grown monoxenically on RFP labeled *Escherichia coli* in a soil slurry. Worms were recovered by density gradient separation and examined using both culture-independent and isolation methods. A 16S rRNA gene survey of the worm-associated bacteria was compared to the soil and to a similar analysis using *Caenorhabditis elegans* N2. Recovered *A. maximus* populations were maintained on cholesterol agar and sampled to examine the population dynamics of the microbiome.

**Results:**

A consistent core microbiome was extracted from *A. maximus* that differed from those in the bulk soil or the *C. elegans* associated set. Three genera, *Ochrobactrum*, *Pedobacter*, and *Chitinophaga*, were identified at high levels only in the *A. maximus* populations, which were less diverse than the assemblage associated with *C. elegans*. Putative symbiont populations were maintained for at least 4 months post inoculation, although the levels decreased as the culture aged. Fluorescence *in situ* hybridization (FISH) using probes specific for *Ochrobactrum* and *Pedobacter* stained bacterial cells in formaldehyde fixed nematode guts.

**Conclusions:**

Three microorganisms were repeatedly observed in association with *Acrobeloides maximus* when recovered from soil microcosms. We isolated several *Ochrobactrum sp.* and *Pedobacter sp.*, and demonstrated that they inhabit the nematode gut by FISH. Although their role in *A. maximus* is not resolved, we propose possible mutualistic roles for these bacteria in protection of the host against pathogens and facilitating enzymatic digestion of other ingested bacteria.

## Introduction

Symbiotic associations with microorganisms are ubiquitous in invertebrates, occurring in both terrestrial and marine environments. These host-microbe symbioses range from mutualistic interactions that enhance the fitness of both organisms to pathogenic associations that compromise the host. The presence of endosymbiont *Wolbachia* in filarial nematodes is exploited in treatment of the widespread parasitic disease, in which they are associated [1]. In entomopathogenic *Heterorhabditis* and *Steinernema* nematodes, the well characterized association with insect-pathogenic bacteria *Photorhabdus* and *Xenorhabdus* is necessary for the worm life cycle to progress [2]. In this beneficial symbiotic association, the worms infect and release endosymbionts that combine to kill the insect host. The nematodes then reproduce within the insect cadaver while feeding on the endosymbionts, after which the bacteria promote their own transmission among insects using the infective juveniles as vectors [3]–[6]. The importance of microbial symbioses with animals is increasingly being recognized as a major theme in biology and characterization of such associations promises to revolutionize the way we view the biotic world [3], [7].

The microbial associations with entomopathogenic nematodes have been well characterized [8], but there are few reports on microbial symbiosis associated with free-living terrestrial nematodes. With further study of nematodes, we can improve our understanding of the evolution of infectious diseases, including those caused by the many human pathogenic bacteria that reside in soil, and that infect free living eukaryotes in the environment [9]. Nematodes are major components of soil ecosystems and are involved in complex symbiotic, pathogenic, and predator-prey interactions with their microbial community. Efforts to understand the host-microbe interactions in diverse organisms can allow us to gain a better understanding of how these interactions have evolved over time and how conserved the mechanisms are across diverse populations. This in turn can allow for improved development of treatments for microbial disease (i.e. by avoiding targets that are present in mutualistic symbionts) or harnessing mechanisms that symbionts use to protect their hosts from pathogens [10].

In this study, we examined potential bacterial symbioses with the free-living soil cephalobid, *Acrobeloides maximus*. This non-pathogenic bacterivore is common in sandy soils that are subjected to high temperatures and protracted desiccation, including coastal dunes as well as xeric inland habitats. In order to specifically observe interactions between the worms and the microbial community, we employed a mesocosm approach. Large populations of “farmed” worm, grown on *E. coli*, were introduced into small soil slurry mesocosms, and then extracted using a density gradient after a 24 h incubation. During this incubation the nematodes were able to feed extensively on the microbial population and microbial colonization processes could occur, without the worms progressing substantially through their lifecycle (28). To capture all types of symbiotic association, we did not surface sterilize the worms after removal from the mesocosm, but simply washed them to remove loosely associated soil microorganisms. The microbial population associated with *A. maximus* was characterized using both culture-independent and culture-dependent techniques. 16S rRNA gene clone libraries constructed from DNA extracted from the nematode's microbiome identified three main organisms that are consistently associated with this nematode. Two of the bacterial associates were successfully isolated, including an alpha-proteobacterium in the genus *Ochrobactrum* and a sphingobacterium in the genus *Pedobacter*. The third associated organism was another sphingobacterium from the genus *Chitinophaga*. The *A. maximus* derived samples were compared with *Caenorhabditis elegans*, a compost dwelling bacteriovore that has been previously examined [11], and with the bacteria extracted from the bulk soil. The resultant populations from *Caenorhabditis elegans* and from the soil were similar to previous published results [12], and were substantially different from the *A. maximus* derived populations. In addition, fluorescence in situ hybridization (FISH) on fixed worms from a separate experiment showed that *Ochrobactrum sp.* and *Pedobacter sp.* are located in the gut of *A. maximus*. Longitudinal studies of the microbiota of worms maintained on agar plates supplemented only with cholesterol illustrated that the population was stable at the phylum level, but at the genus level the putative symbionts were replaced by other bacteria that may be associated with decline in worm viability. The loss of the two major symbionts as the worm culture ages suggests an active maintenance process. Based on the location of the bacterial symbionts, we suggest that they may provide protection against pathogenic bacteria in the environment or aid in digesting the diverse microbiota, or both.

## Materials and Methods

### Nematode cultivation and harvest

*Acrobeloides maximus* and *Caenorhabditis elegans* were grown monoxenically on *Escherichia coli* S17-1 containing a constitutive red fluorescent protein (RFP) expression plasmid using nutrient-free agar plates supplemented with cholesterol at a concentration of 1 µg/ml that is necessary to maintain viable worms [13]. After growth to high density on agar plates worms were harvested in water. Worm populations were counted directly and diluted to 10^4^ worms/ml for soil exposure. The *C. elegans* population was grown from bleach sterilized eggs, and the *A. maximus* cultures used were provided from long-term stock cultured maintained by one of the authors (PDL).

### Rhizosphere soil collection and exposure

Rhizosphere soil from wild sunflowers at the California State University, San Bernardino (CSUSB) Nature Preserve was collected in sterile conical tubes. This soil is similar to the environments from which these worms were originally collected (P. deLey, unpublished). The samples were then stored at 4°C until processing. Samples were weighed and subsequently exposed to worms by adding 1 ml of the worm slurry per gram of soil, in batches containing approximately 5 g of soil. For initial population studies, soil samples were exposed to *A. maximus* in triplicate, along with a soil sample that contained *C. elegans* as well as one containing 1 ml sterile water g^−1^ soil. Samples were incubated at room temperature for 24 h prior to worm recovery. This time period was chosen to allow the worms to move and eat in the soil slurry without allowing either species to move through a significant portion of their life cycle (4 d for *C. elegans*, 8 d for *A. maximus* [28]). We also estimated that this would be sufficient time for bacterial colonization to occur. After 24 h, the worms were harvested by centrifugal flotation using MgSO_4_ heptahydrate added to the samples to a final concentration of 409 g L^−1^ [14]. The soil was pelleted at 1800× *g*, and the supernatant containing the worms as well as other organisms from the soil was removed. This solution was filtered on a 0.2 µm filter, and the worms retained on the filter were then collected in 3 ml of sterile water. Aliquots of this slurry were used for long-term culture studies. The slurry was washed 2× with water to remove loosely associated bacteria, and then preserved for DNA extraction and analysis by freezing at −20°C. This collection method was repeated for FISH probing of exposed worms using identical exposure and recovery techniques.

### Long-term culture of *A. maximus*

Rhizosphere exposed nematodes were grown on cholesterol supplemented plates (described above) at room temperature, with no other food source. At selected time points (3 wks, 2, 4 and 6 months), the nematodes were collected for analysis and subcultured for further observation. These time points allowed for several generations to emerge on the freshly cultured plates (*A. maximus* generation time at room temp is 8 d [15]). The plate material was collected using sterile PBS, and washed as described above to remove free-living bacteria. The continuing culture was seeded with 50 µl of the washed worm suspension, and the remainder was frozen for subsequent DNA analysis.

### DNA extraction and PCR analysis

DNA was extracted from the rhizosphere soil along with *A. maximus* and *C. elegans* rhizosphere exposed soils and the long term cultures using the FastDNA Spin Kit for Soil (Qbiogene). The resultant DNA from all samples was then individually amplified using the GenomiPhi V2 DNA Amplification Kit (GE Healthcare Life Sciences). This amplified DNA was used as the template DNA for PCR analysis. Near full length 16S rRNA gene fragments were amplified using the 27F and 1492R universal primers [16].

The PCR reaction conditions were as follows; 1× concentration of GoTaq Green PCR Master Mix (Promega), 0.5 µm of both 27F and 1492R primers and at least 20 ng of template DNA in a 50 µl reaction, using the following method: 95°C for 5 min then 30 cycles of 94°C for 30 sec, 55°C for 30 sec, 72°C for 1 min, followed by a final extension step for 10 min at 72°C. PCR products were verified using agarose gel electrophoresis stained with ethidium bromide and then purified using the Wizard SV Gel and PCR Clean-up System (Promega).

### 16S rRNA gene library construction, sequencing and analysis

The PCR products were ligated into the pCR 2.1-TOPO vector and cloned with the TOPO TA Cloning Kit using One Shot TOP10 Electrocompetent *E. coli*. Sets of 94 clones from each of the three *A. maximus*-rhizosphere exposed samples, 94 clones from the *C. elegans*-rhizosphere exposed sample and 94 clones from the rhizosphere sample were sequenced (Laragen, CA). In addition, 94 clones from each of the four time points of the long-term culture studies were also sequenced. Samples from the two separate long term culture plates (94 clones each) were sequenced for the initial (3 wk) time point, but based on the very similar results from these samples only one plate was sequenced for the remaining long term culture time points. All of these clones were sequenced using the M13 for primer site in pCR2.1, and a single direction read was used for initial phylogenetic analysis. All sequences were trimmed using Geneious sequence analysis software (v. 3.7.1). The trimmed sequences from all of these experiments are available in **Data S1**. The identification of OTUs was performed using the Greengenes pipeline (http://greengenes.lbl.gov) using the Ribosome Database Project (RDP) phylogeny (97% cutoff) [17], [18]. The batch sequence files were uploaded in FASTA format, and checked for chimeric sequences using the Bellerophon program [17]. Since the sequences were derived from primers in the vector, and the cloning was not directional, the sequences were first aligned against the prokMSA 16s sequence database using the NAST algorithm [18]. These alignments are provided in **Data S2**. After this alignment was completed the aligned set was compared to the same database using the greengenes classifier. This algorithm finds the nearest neighbors to the input sequences using Simrank, and subsequently calculates the sequence divergence from the nearest neighbor to determine Operational Taxonomic Unit (OTU) assignment (http://greengenes.lbl.gov/cgi-bin/nph-classify.cgi) [17]. The classification output is available in **Data S3**. The Shannon-Weiner index of diversity and Shannon-Weiner evenness were calculated using tools found at Chang Bioscience based on the Greengenes assigned OTUs. The sample Acro2 was subsequently resequenced in the reverse direction for more fine-grained analysis of the *Pedobacter*, *Ochrobactrum*, and *Chitinophaga* populations. For these groups, the nearly full length 16S rRNA gene sequences were trimmed and aligned in Geneious. Sequences from Genbank were gathered in each genus and phylogenetic trees were constructed using Mr. Bayes [19] with the HKY85 nucleotide substitution model [20]. All of the Bayesian trees were constructed using the Markov chain Monte Carlo algorithm with 1,100,000 chain length and 100,000 burn-in length. The input to the model was the ClustalW alignment of the nucleotide sequences, and the tree branch lengths were unconstrained. All of these analyses were performed using the Geneious Pro 5.3.4 (Biomatters, Ltd, New Zealand) platform for sequence analysis. For the *Ochrobactrum* tree, *Rhizobium leguminosarum* bv. trifolii WSM597 was used as the outgroup, along with *Ochrobactrum lupine*, *Ochrobactrum anthropi* strains SRK-5 and ATCC 49188, *Ochrobactrum* sp. BH3, *Ochrobactrum cystisi* SRK-3, *Ochrobactrum* sp. TD, and *Ochrobactrum* sp. B2 BBTR46. The isolate from this study, *Ochrobactrum* strain W2P, was also included in this analysis. The tree built using Acro_2 sequences from the genus *Chitinophaga* included *Flavobacterium johnsoniae* UW101 as the outgroup, along with *Chitinophaga terrae*, *Chitinophaga japonensis* strain 758, *Chitinophaga ginsengisegetis*, *Flexibacter sp.* MG5, *Chitinophaga* sp. LA7, and *Pedobacter heparinus* to help identify phylogenetic clusters. The tree built to examine the *Pedobacter* isolates included the isolated *Pedobacter* strain API, as well as *Chitinophaga terrae* as the outgroup. Other species included to augment phylogenetic analysis were from *Pedobacter saltans*, *Pedobacter heparinus*, *Pedobacter metabolipauper*, *Pedobacter duraquae*, *Pedobacter sp.* W48, and *Pedobacter cryoconitis*. DGGE derived sequences from previous work on *Heterodera* soybean cysts were also included in the analysis of *Chitinophaga* and *Pedobacter* [21]. All the nearly full length Acro2 sequences have been deposited into Genbank (Accession #KC110895-KC110987).

### Isolation of associated bacteria

During the initial culture of bacterial associates from worms recovered from the microcosms, individual worms were transferred on to rich medium containing 5 g L^−1^ yeast extract (YE) or water agar. After the worms had crawled on the plates for up to 24 h, the worms were removed and the bacteria allowed to grow. Additional inoculations from eggs laid on long term culture plates were also performed, as well as from worms in varying stages of decay. Colonies were also observed on the water agar, which were picked for isolation onto water agar and then subsequently grown in isolation on YE plates. All isolates were subsequently identified by PCR amplification of their 16s rRNA genes and analysis using the Greengenes pipeline (see **Data S3**).

### Fluorescent in-situ hybridization of the two bacterial symbionts

Cultivation and exposure of nematodes was carried out as described above. After 24 h, the worms were collected as before along with worms collected from agar plates with no soil exposure. The worms were immediately fixed in paraformaldehyde and stained with oligonucleotide probes designed to specifically anneal to either the *Ochrobactrum* or *Pedobacter* 16S rRNA based on the database available at probeBase [22] (http://www.microbial-ecology.net/probebase/). The probe specific to *Pedobacter sp.* PED672 [23] was linked with sulfoindocyanine chromophore Cy3 and the Ochro3 probe specific to *Ochrobactrum sp.* [24] was linked to Cy5. FISH staining procedures were followed as previously described by Vandekerckhove et al. [25]. DIC and fluorescence images from fixed samples were collected using a Zeiss Axioplan2 equipped with a CoolSnapFX cooled ccd camera and ImageProExpress 2.0 capture software.

## Results

### 16S rDNA analysis of extracts

**Figure 1:**
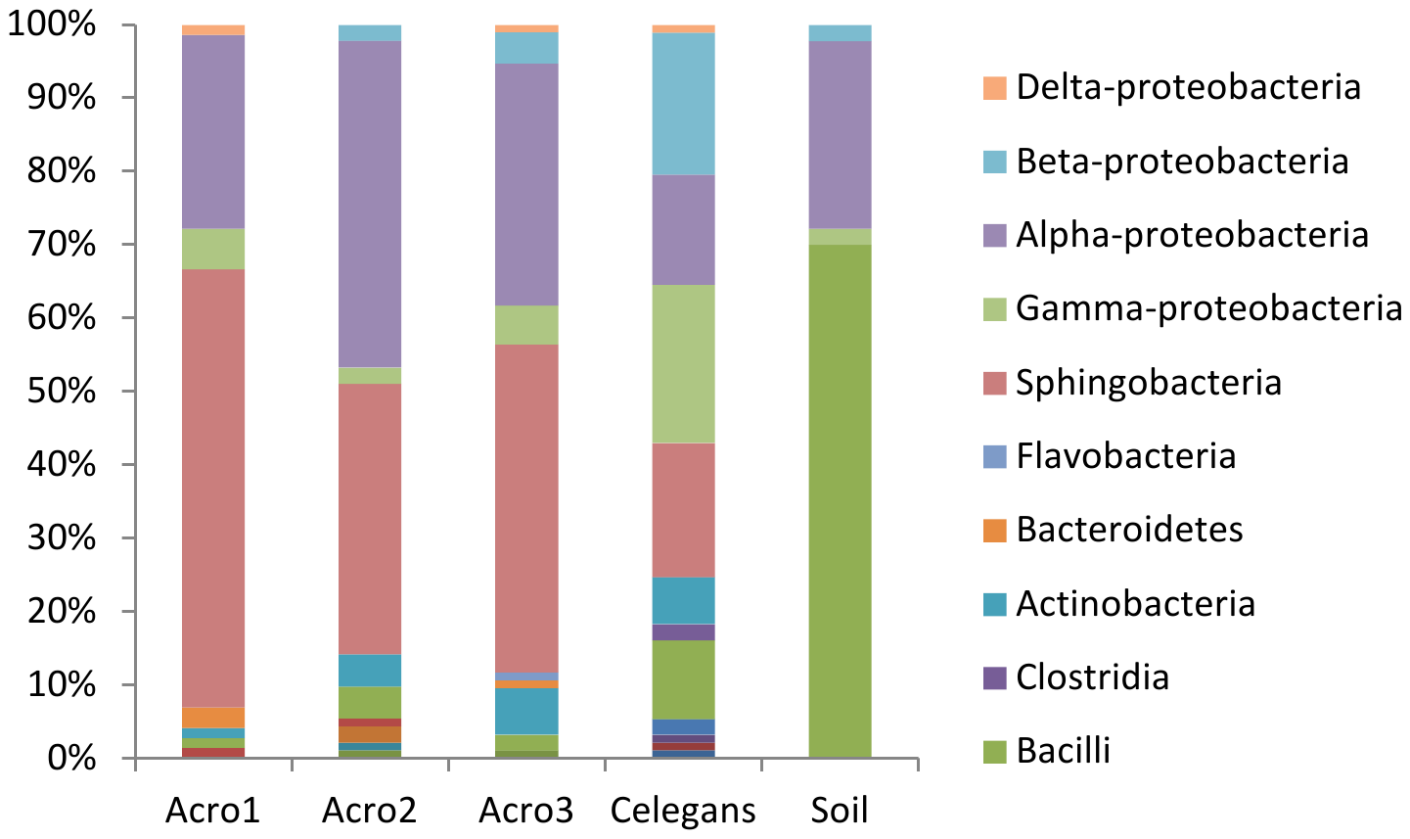
A consistent bacterial population is associated with soil exposed *A.* *maximus* nematodes. Samples isolated from *A. maximus* (N = 72, 93, 92 for Acro1-3) are dominated by Sphingobacteria and α-Proteobacteria after 24 h soil exposure. The bacteria from the identically treated soil sample (N = 94) contain a much higher percentage of Bacilli, and those associated with *C. elegans* after 24 h soil exposure (N = 94) are more broadly distributed across taxa.

**Figure 2:**
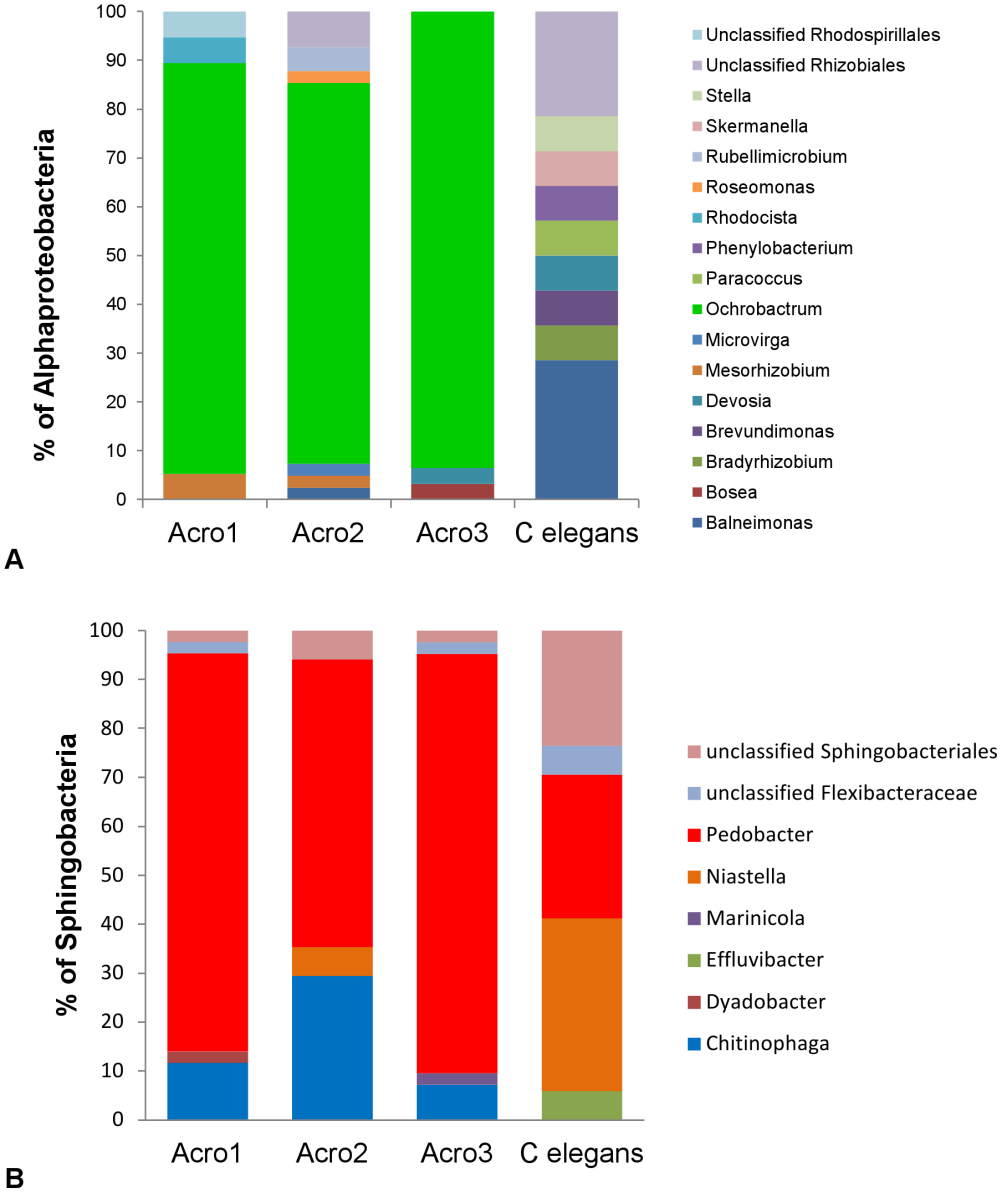
Within the dominant subgroups the *Acrobeloides maximus* populations were consistent and limited compared to *C.* **elegans**** **associated bacteria.** **** **A**) The alpha-proteobacteria within *A. maximus* were predominantly Ochrobactrum. **B**) The Sphingobacteria within *A. maximus* were predominantly *Pedobacter*, with a consistent representation of *Chitinophaga* at a lower level. The *C. elegans samples* were more diverse at the genus level (both panels).

After exposure to soil for 24 h, worms were recovered by density dependent extraction, washed to remove free-living bacteria, then examined by culture-independent methods. A high taxonomic level analysis of these populations based on the amplification of 16S rRNA gene sequences from these samples is shown in **Figure 1**. The microbiota of the *A. maximus* samples collected were similar in the three separate extractions and dominated by alpha-Proteobacteria and Sphingobacteria (**Fig. 1**). The microbiota from *C. elegans* and the soil control were extracted using similar approaches, and found to be similar in structure to previously published reports, but were much different when compared to the *A. maximus* associated population [11], [26] (**Fig. 1**). Diversity index calculations and analysis of population structures in the samples reveal marked differences (**Table 1**). The semi-arid soil from the sunflower rhizosphere was dominated by *Paenibacillus* and alpha-Proteobacteria (**Fig. 1**), while the *C. elegans* derived microbiota was considerably more diverse than either the soil or the *A. maximus* samples (**Table 1**). Inspection of sequences within the two major clades from the *A. maximus* samples, indicated there was a single dominant genus in each group, that was not detected in the soil samples (data not shown)or in samples from *C. elegans* (**Fig. 2**). The feeding strain of *E. coli* which constitutively expresses RFP was never recovered from any of the samples, nor was red fluorescence observed in any subsequent experiments (data not shown).

**Table 1:** Shannon-Wiener Population Statistics for Microcosm Samples.

	Acro 1	Acro 2	Acro3	*C. elegans*	Soil
SW Diversity Index	**1.78**	**2.26**	**1.96**	**3.41**	**1.31**
SW-Evenness	**0.63**	**0.71**	**0.64**	**0.9**	**0.53**

### Co-cultures

**Figure 3:**
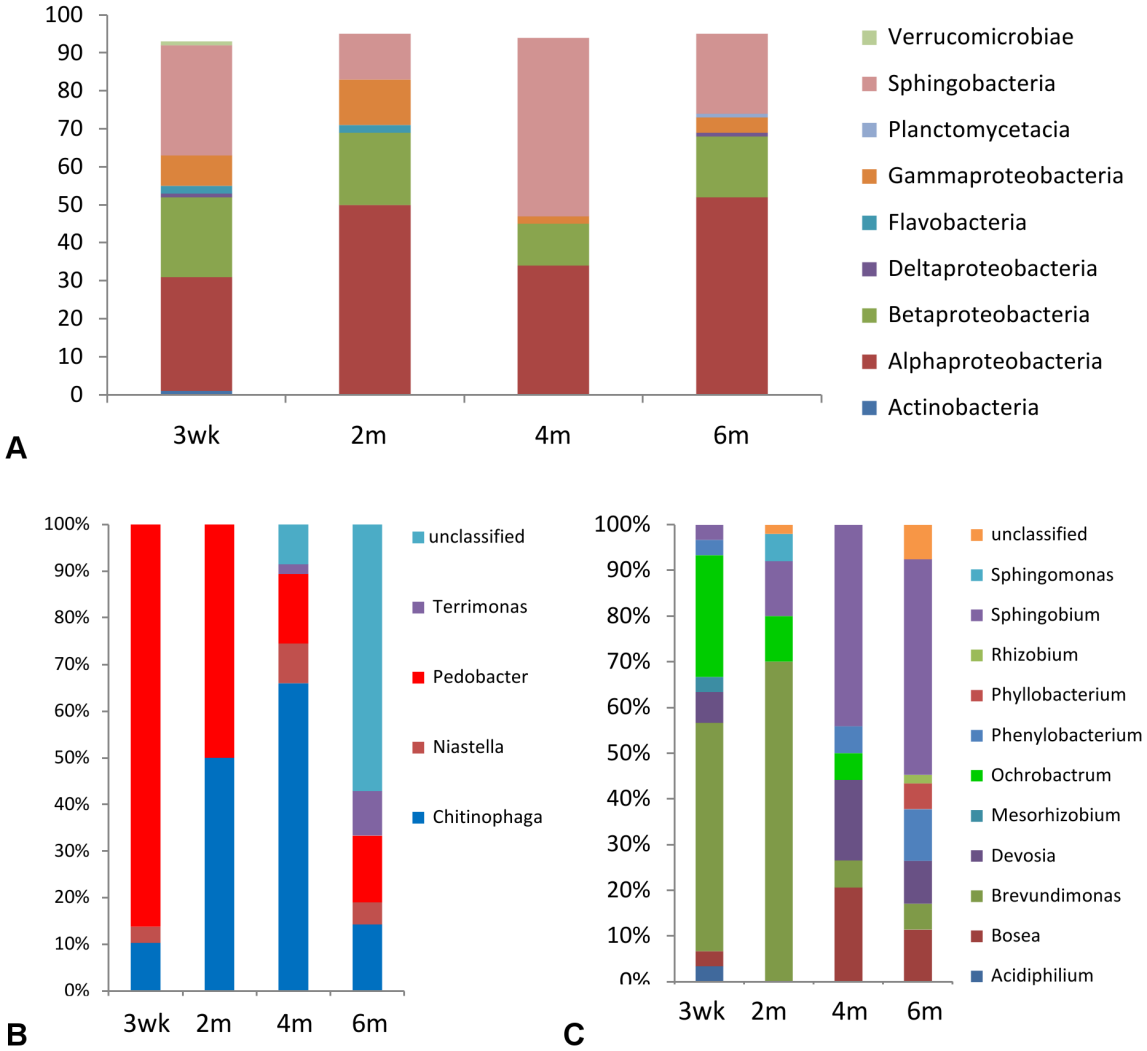
Long term culture experiments sampled at 3 wks, 2 m, 4 m, and 6 m post inoculation appear to show stability at the phylum level (A) but reveal shifts in population as the worm population ages at the genus level (B,C). At 6 m post exposure the initially dominant clades have been supplanted in both the Sphingobacteria (**B**) and Alpha-Proteobacteria (**C**).

Worm co-cultures with bacteria from the soil were maintained in duplicate for a period of six months with periodic collection and subculture. When the microbiota of the 3 wk co-cultures were examined by 16S rRNA methods, a stable population was observed with a simiIar make-up to the samples recovered from soil exposure (**Fig. 3**, results from one long term culture shown). As the co-cultures were extended over 6 months, the overall populations shifted away from the original structure (**Fig. 3A**), although members of *Pedobacter* and *Chitinophaga* were present throughout the experiment (**Fig. 3B**). *Ochrobactrum* was present in all but the final sample (**Fig. 3C**). The worm populations after 6 months were generally more likely to contain quiescent larvae and unhatched eggs, but relatively few adults (data not shown), and the bacterial population was dominated by *Sphingobium* and unclassified Sphingobacteria (**Fig. 3C**). In the early stages of the long-term culture, a substantial *Brevundimonas* contingent was observed, and an increasing fraction of the Sphingobacterial population was *Chitinophaga*, peaking at 4 months post-inoculation (**Fig. 3C**).

### Isolation and culture

Isolates were picked from the co-cultures at various time points by direct bacterial isolation or by isolating individual worms on different media. These bacterial isolates were identified by 16S rRNA gene sequencing and taxonomic analysis (**Table 2**). Isolates from the genus *Bacillus* were recovered from apparently dead *A. maximus* eggs and worms. Most of the other isolates, including *Ochrobactrum*, were recovered by picking individual live worms onto YE medium and subsequent isolation. To isolate *Pedobacter*, it was necessary to transfer worms onto fresh agar plates containing no potential nutrients for bacterial growth, and isolate microcolonies that formed. Subsequent microcolony isolation and analysis by 16S rRNA gene sequencing resulted in the cultivation of a *Pedobacter* isolate. The *Pedobacter* and *Ochrobactrum* isolates were cultivated axenically.

**Table 2:** Identified Isolates from *A. maximus* microcosm and long term culture.

Accession	Isolate name	OTU^a^	Source(s)
KC687078	*Pseudomonas sp. JPB-PO4*	1813	Nematode long term culture and isolation on YE
KC687079	*Massilia sp. PO12*	396	Nematode long term culture
KC687080	*Pseudomonas sp. PO7*	1813	Nematode associated isolation on YE
KC687081	*Pedobacter sp. API*	534	Nematode long term culture, oligotrophic medium
KC687082	*Salmonella sp. JPunk1-2*	1607	Nematode associated isolation on YE
KC687083	*Pseudomonas sp. JPunk3-1*	1813	Nematode associated isolation on YE
KC687084	*Brevundimonas sp. JPunk3-2*	1066	Acrobeloides stock plate
KC687085	*Ochrobactrum sp W2P*	1108	Nematode associated isolation on YE
KC687086	*Bacillus sp. JPB-PO2*	758	Dead worm in long term culture
KC687087	*Bacillus sp. JPB-PO3*	758	Nematode associated isolation on YE
KC687088	*Achromobacter sp. JPB-PO7*	1317	Nematode associated isolation on YE

a OTU assigned using RDP phylogeny. Isolates assigned to the same OTU were verified to be distinct using direct sequence comparison (bl2seq).

### Phylogenetic analysis

**Figure 4:**
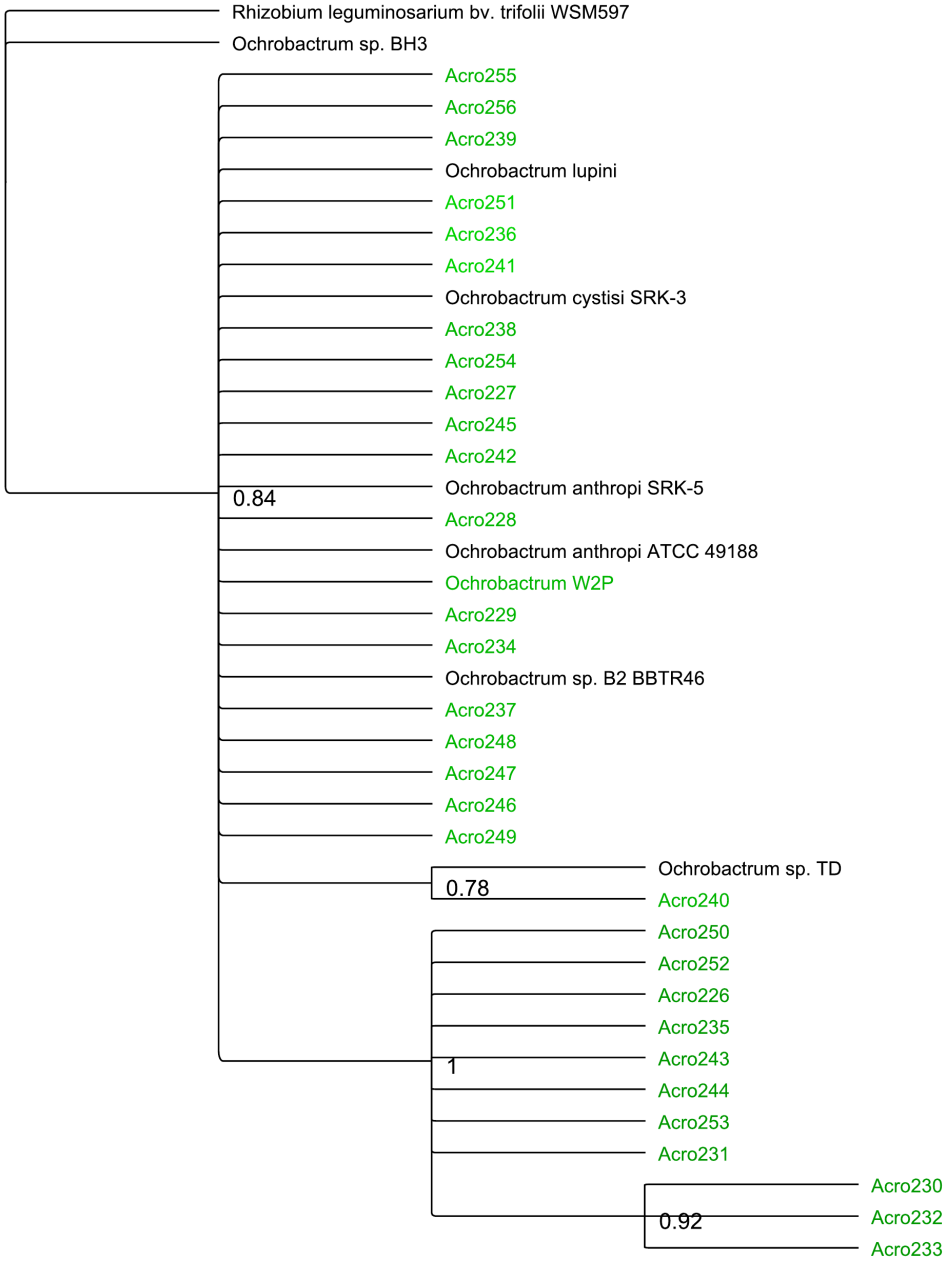
Phylogenetic tree created using Bayesian inference on the Ochrobactrum 16S rDNA sequences obtained from sample Acro2, the isolated *Ochrobactrum* sp., and several related sequences from the literature (see Materials and Methods). *Rhizobium leguminosarum* bv. trifolii WSM597 was used as the outgroup for this analysis.

One of the three sample sets from *A. maximus* inoculation into soil (Acro2) was used for further phylogenetic analysis based on near full length 16S rRNA gene sequences generated in a second set of sequencing reactions. . Sequences from this set were identified using Greengenes [17], [18] and subsequently the sequences from the genera *Pedobacter*, *Ochrobactrum*, and *Chitinophaga* were analyzed in further detail. Based on BLAST search results, sequences from Genbank were used to determine the phylogenetic relationships. Additional sequences (clone A and clone B) were identified from previous work on bacteria associated with the Soybean Cyst Nematode *Heterodera* [21]. Phylogenetic trees (**Fig. 4** **,** **5** **,** **6**) based on Bayesian inference was created using the sequences for each genus, with additional sequences from each genus chosen from the published literature.

**Figure 5:**
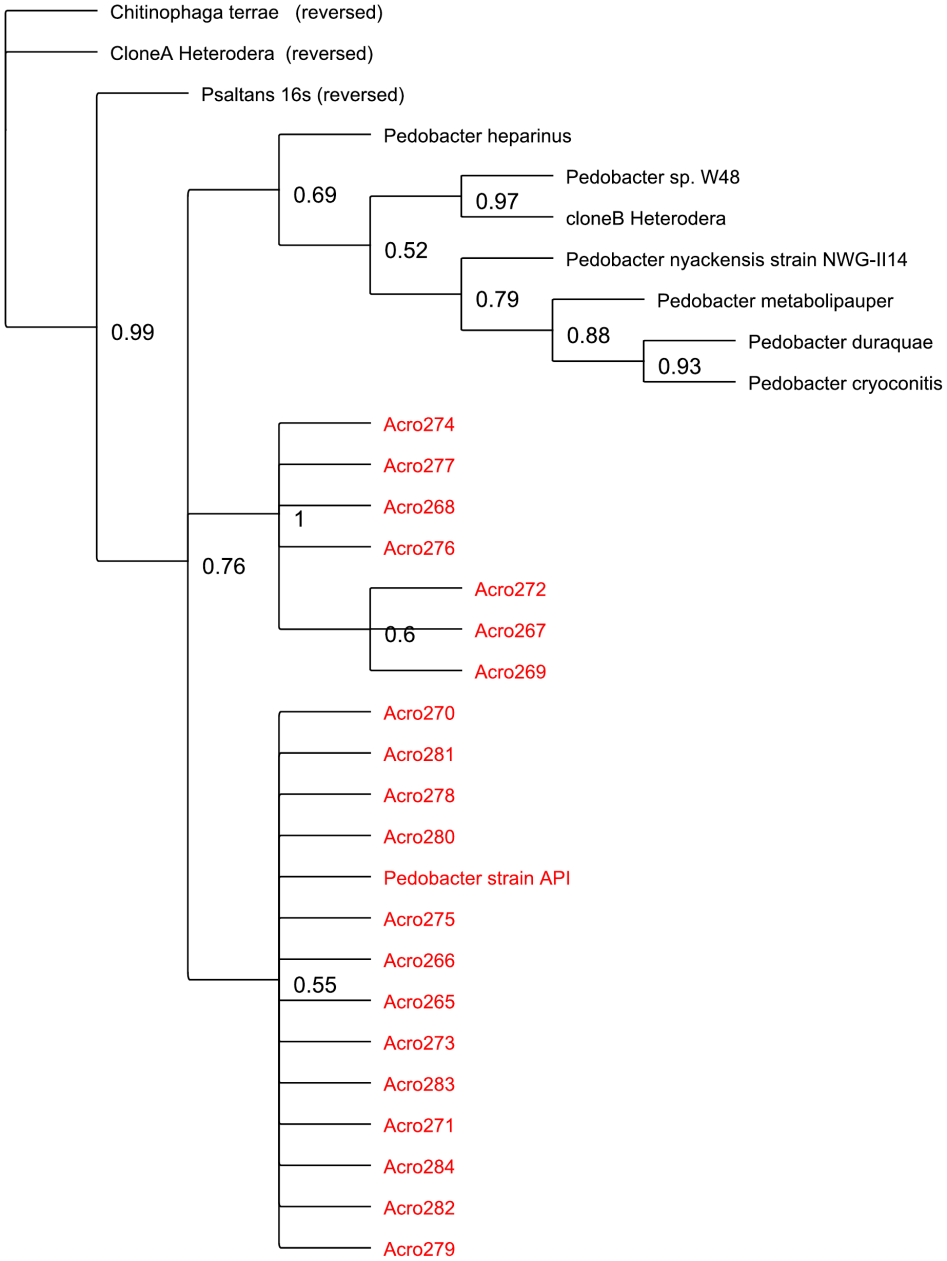
Phylogenetic tree created using Bayesian inference on the *Pedobacter* 16S rDNA sequences obtained from sample Acro2, the isolated *Pedobacter* sp., and several related sequences from the literature (see Materials and Methods). Clone A (Heterodera) and Clone B (Heterodera) are partial sequences from Nour et al [21]. *Chitinophaga terrae* was used as the outgroup for this analysis.

**Figure 6:**
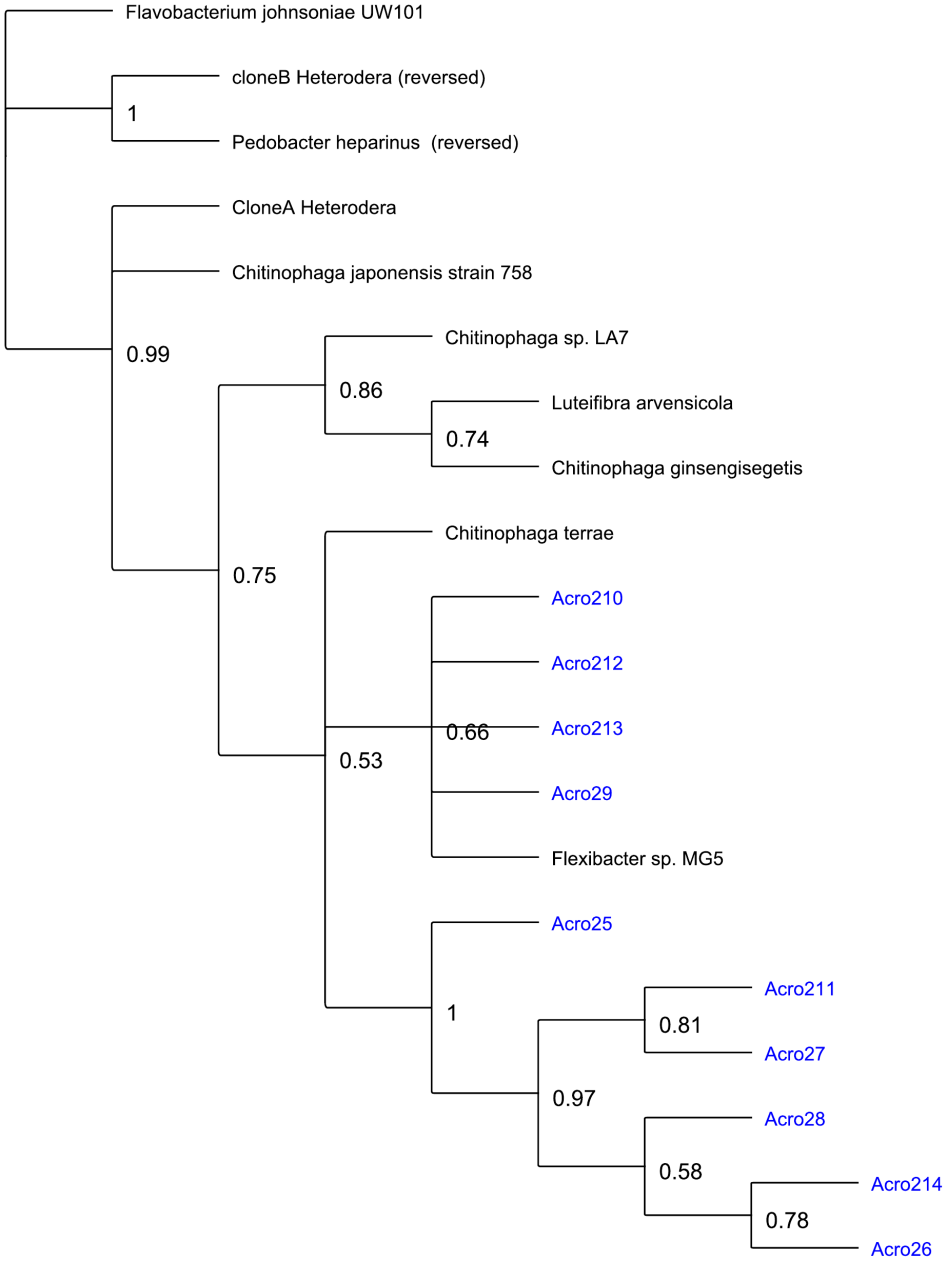
Phylogenetic tree created using Bayesian inference on the *Chitinophaga* 16S rDNA sequences obtained from sample Acro2, and several related sequences from the literature (see Materials and Methods). Clone A Heterodera and Clone B Heterodera were previously identified microbial sequence tags recovered from the Soybean Cyst Nematode [21]. *Flavobacterium johnsoniae* UW101 was used as the outgroup for this analysis.

#### FISH

**Figure 7:**
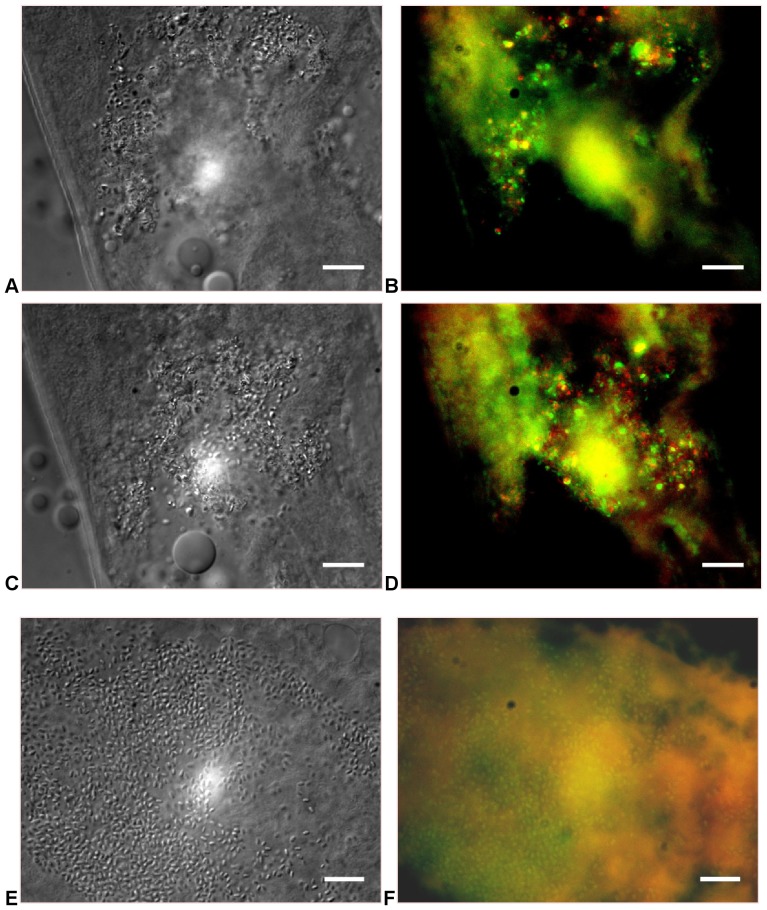
Fluorescence *in situ* hybridization to identify *Ochrobactrum* (green) and *Pedobacter* (red) in the gut of formaldehyde fixed *A.* *maximus* after recovery from soil microcosm. Samples from soil microcosms (**A–D**) and monoxenic culture on *E. coli* DH5μ (**E, F**) were imaged by DIC (**A,C,E**) or epifluorescence (**B,D,F**). Scale bar = 10 microns.

When worms were extracted from soil after 24 h of exposure, and probed for the presence of *Ochrobactrum* and *Pedobacter* using FISH, signals corresponding to both genera were identified in the guts of the worms (**Fig. 7 A–D**). Control experiments using the cultured strains of *Ochrobactrum*, *Pedobacter*, (not shown) showed no cross reactivity of the probes under the fixation and hybridization conditions used and no background staining was seen in worms fed only *E. coli* (**Fig. 7E and F**). Organisms from both genera were found in the guts of the worm, and rarely identified in any other areas of the organisms consistently.

## Discussion

Sequences associated with *Ochrobactrum*, *Pedobacter*, and *Chitinophaga* were consistently identified in all DNA samples isolated from the nematode *Acrobeloides maximus* recovered from soil microcosms. Our experiment did not include a surface sterilization step, in order to include all potential types of microbial association, not only gut symbioses and endosymbioses. The sequences from the three putative symbiont genera were substantially enriched in *A. maximus* derived samples compared to either direct sampling of the soil or similar sampling from soil exposed to *Caenorhabditis elegans*, suggesting that these are clade specific associations. Further investigation in long-term cultures showed that these organisms persisted in co-culture with the worms on agarose plates where no external nutrients are provided to either the worms or the soil derived microbiota. These samples were fairly stable at the phylum and subphylum level, but within the dominant groups (alpha-proteobacteria and sphingobacteria) the populations shifted substantially over time. This is likely due to dissipation of nutrients from the soil over time in these cultures. Isolations on low nutrient and high nutrient media were successfully undertaken, and the subsequent experiments using FISH to identify *Ochrobactrum* and *Pedobacter* showed that both of these bacteria were able to colonize the nematode gut.

It is important to note that other work characterizing the microbiota of nematodes has provided similar results. Studies of wild *C. elegans* identified both *Ochrobactrum* and *Pedobacter* as worm associated genera [11]. In addition, a study on the soybean root cyst nematode *Heterodera* also identified bacteria from these two genera, although the identification was not made in that article because of the limited sequence data available at the time [21]. The investigators in that work were also able to cultivate *Pedobacter* from the soybean cyst. Recent investigations with the plant parasitic nematode *Bursaphelenchus* [27] also identified *Pedobacter* as an associated bacterium, indicating that the relationship may be a broad association with many nematodes rather than specific to *A. maximus*.

It is apparent from our work and others that laboratory bred *C. elegans* (N1) is unusually unselective in its microbiota. This may have relevance to experiments using nematodes as models for infectious disease. The strong and repeatable shift of the *A. maximus* microbiome away from the soil microbial structure indicates that the formation of the microbiome occurs rapidly when the worms are introduced into the soil, and is a result of specific host-microbe interactions. We also note that the *E. coli* S17-1 that was used to raise all of the worms monoxenically before inoculation into the soil was rapidly displaced in both *C. elegans* and *A. maximus*. The long-term culture studies indicated that the external conditions also played a role in determining the structure of the microbiome, Many of the worms in the long term cultures entered a quiescent state, and others died. Many unhatched eggs were also observed to accumulate on these plates, some of which were eventually overgrown and apparently decomposed by the bacterial population. Being highly adapted to xeric soils, we hypothesize that *A. maximus* eggs probably hatch differentially after one or more external cues, probably as a result of bet-hedging mechanisms.

When we examine the phylogenetic relationships within the putative symbiont cultures, and compare the sequences recovered to previously published representatives of these genera, we see several different patterns in the different species. The observed polytomy in the *Ochrobactrum* tree, with only a few sub-genus level clusters, suggests a limitation of 16s rRNA based phylogeny (**Fig. 4**). The *Chitinophaga* and *Pedobacter* trees indicate that the sequences from this work form a distinct clade from previously identified species (**Fig. 5** **,** **6**). When examining the literature on nematode associated bacteria, we found that previous work using DGGE and sequencing with *Heterodera* as the host nematode [22] had identified several Flavobacteria (clone A and clone B *Heterodera*, **Fig. 5** **,** **Fig. 6**). When we added these clones to our trees, they clustered with *Chitinophaga* and *Pedobacter* respectively, indicating that there may be some common themes in these nematode-bacteria associations.

The associations we observed were stable and reproducible, but not necessarily impervious to shifts when cultured in the lab over the long term. We were not able to culture *Chitinophaga* from our enrichments, nor were we able to find a suitable probe to examine the fixed worms using FISH the presence of this particular microbe. However, we were able to repeatedly recover *Ochrobactrum* and *Pedobacter* in association with *A. maximus* both in culture based and culture independent tests, and using FISH we were able to observe these bacteria in the nematode gut. Based on these observations, we hypothesize that the host-microbe relationships are mutualisms, although commensalism is also certainly possible. Two hypotheses immediately suggest themselves: the putative mutualists might help the worm digest its food through secretion of toxins, antibiotics, or digestive enzymes, or they might protect against invasion by pathogenic bacteria. Strains of *Pedobacter* have been associated with antifungal and probiotic activities in various circumstances [28], while some reports have proposed that *Ochrobactrum* protects entomopathogenic nematodes from pathogens [29]. The widespread association of these particular bacteria with several different and diverse nematode species suggests an important evolutionary relationship that merits further study to determine the role of symbiosis in the behavior and ecology of soil nematodes.

## Supporting Information

Data S1 **Trimmed sequence files used in phylogenetic analysis from soil microcosm and long term culture samples.** All were sequenced using plasmid primer binding sites (M13F or M13R) and the plasmid sequence was trimmed away. The sequences were trimmed at the 3′ end at the first ambiguous nucleotide.
(ZIP)

Data S2 **Sequence alignments for each data set using the NAST Alignment algorithm.**
(ZIP)

Data S3 **Classification algorithm output for all samples from the Greengenes classify algorithm.**
(ZIP)
